# An Assessment of the Sensory Function in the Maxillofacial Region: A Dual-Case Pilot Study

**DOI:** 10.3390/s25113355

**Published:** 2025-05-26

**Authors:** João Maia Aguiar, José Machado da Silva, Carlos Fonseca, Jorge Marinho

**Affiliations:** 1 INESC TEC—INESC Technology and Science, 4200-465 Porto, Portugal; jms@fe.up.pt; 2 Faculdade de Engenharia, Universidade do Porto, 4200-465 Porto, Portugal; cfonseca@fe.up.pt; 3 LAETA/INEGI, Institute of Science and Innovation in Mechanical and Industrial Engineering, 4200-465 Porto, Portugal; 4 Instituto Português de Oncologia do Porto FG, EPE (IPO-Porto), 4200-072 Porto, Portugal; jmarinho@ipoporto.min-saude.pt

**Keywords:** trigeminal somatosensory-evoked potentials, dental implants, electroencephalography, dental proprioception, neurostimulation, pilot project

## Abstract

Trigeminal somatosensory-evoked potentials (TSEPs) provide valuable insight into neural responses to oral stimuli. This study investigates TSEP recording methods and their impact on interpreting results in clinical settings to improve the development process of neurostimulation-based therapies. The experiments and results presented here aim at identifying appropriate stimulation characteristics to design an active dental prosthesis capable of contributing to restoring the lost neurosensitive connection between the teeth and the brain. Two methods of TSEP acquisition, traditional and occluded, were used, each conducted by a different volunteer. Traditional TSEP acquisition involves stimulation at different sites with varying parameters to achieve a control base. In contrast, occluded TSEPs examine responses acquired under low- and high-force bite conditions to assess the influence of periodontal mechanoreceptors and muscle activation on measurements. Traditional TSEPs demonstrated methodological feasibility with satisfactory results despite a limited subject pool. However, occluded TSEPs presented challenges in interpreting results, with responses deviating from expected norms, particularly under high force conditions, due to the simultaneous occurrence of stimulation and dental occlusion. While traditional TSEPs highlight methodological feasibility, the occluded approach highlights complexities in outcome interpretation and urges caution in clinical application. Previously unreported results were achieved, which underscores the importance of conducting further research with larger sample sizes and refined protocols in order to strengthen the reliability and validity of TSEP assessments.

## 1. Introduction

The buccal cavity and its functions are of significant importance to humans in various physiological and social domains, including ingestion, articulation, and postural regulation, as well as verbal and facial expression [1]. Each anatomical component of the oral cavity plays a unique and vital role in ensuring effective functioning, and all of them work together to achieve different purposes.

The mouth functions optimally when input is received from various sensory organs and specific muscles are properly controlled [2]. For example, speaking involves the respiratory, phonatory, and articulatory systems. During speech production, air leaves the lungs, enters the windpipe, and reaches the larynx, where it is modulated as it passes through the vocal folds (or vocal cords). When the vocal folds come together, they vibrate as the air passes, producing sounds. The parts of the vocal tract shape these sounds into the words that make up speech. All of these functions are controlled by the brain, where a complex network is responsible for the speech-forming processes, from putting thoughts into words to forming a comprehensible sentence and stimulating different muscles to produce the correct sounds. In addition, the sensory organs, including the ears, which detect the sound produced, and the tongue and mouth, which detect the position and movement of the articulators, are involved in this process to transmit information to the brain.

Masticatory function involves the teeth, chewing muscles, temporomandibular joints, and the nervous system. Chewing starts with biting, where food is cut or crushed by the teeth. Saliva mixes with the food to form a bolus. The tongue then pushes the bolus back for swallowing. Chewing muscles move the jaw, while sensory receptors in the teeth, tongue, lips, and mouth detect features like size, hardness, texture, temperature, pain, and taste. Teeth, in particular, can sense pressure, pain, and temperature changes [3]. This information is conveyed to the brain by superior and inferior alveolar nerves, or terminations of the trigeminal nerve, which provide general sensation to most of the oral cavity [4].

Periodontal mechanoreceptors are specific afferent neurons located in the teeth’s periodontal ligaments, the connective tissue that attaches the tooth to the alveolar bone, which sense and transmit information about the force, direction, and duration of tooth loads to the central nervous system [2]. This information is essential for controlling oral motor behaviors, such as biting, chewing, swallowing, and speaking. Periodontal mechanoreceptors also help to protect the teeth and the jaw from excessive or harmful forces by modulating the activity of the masticatory muscles and the salivary glands.

### 1.1. Consequences of Tooth Loss

Proprioception is the body’s self-conscious awareness of its movement, strength, and position. Oral proprioception refers to the sensory feedback process that helps one to perceive the position, movement, and force of the mouth and its parts (tongue, lips, teeth, etc.).

Buccal mechanoreceptors are found in the periodontal ligament, alveolar mucosa, gingiva, bone, periosteum, and tongue. Periodontal mechanoreceptors contribute to the precise execution of complex muscle movements, including the alternating contraction and relaxation required to chew and bite. They also act as gyroscopes for the brain. The absence of this feedback can lead to postural disorders [5], increased risk of certain cancers, dementia, and stress disorders [3,6,7,8]. In addition, excess weight puts extra strain on the bone, which can lead to unexpected tooth loosening. Excess bone stress can also result in a lack of bone remodeling capacity and eventual pathological overload, leading to bone loss.

Tooth loss is a serious condition that affects your physical and mental health. Complete edentulism is estimated to affect approximately 23% of the world’s adult population over the age of 60 [9]. Losing teeth can lead to the following consequences:

- Difficulty eating certain foods, which can affect nutrition and digestion, as well as the enjoyment of food;
- Higher self-consciousness over appearance, which can make one feel less confident and attractive, leading to avoiding social situations or interactions that involve smiling or talking, as well as impairing communication, comprehension skills, and self-esteem;
- Increased risk of damage to surrounding teeth, such as misalignment, crowding, or tilting of the teeth, which can make them more prone to decay, fracture, or infection;
- Speech problems, such as the ability to pronounce certain sounds and words;
- Greater likelihood of gum disease and cavities due to exposing more of the gum tissue and the roots of the remaining teeth;
- Affects the density and shape of the jawbone, which supports the teeth and maintains the facial structure.

Following tooth loss, whether through aging or illness, the remaining periodontal ligaments disintegrate and disappear, reducing every sense of biting and chewing strength. The frequent failure and deterioration of dental prostheses can be attributed to this loss of information [10,11,12]. Additionally, this factor contributes to numerous complaints, such as headaches, back pain, and poor posture, reported by patients when chewing on dental implant prosthesis fixtures [13].

Most problems associated with fixed prostheses, including ceramic fracture, unsecured cement or screws, abutment screw loosening, peri-implantitis, implant-related bone loss, and premature implant loading failure, are due to biomechanical factors such as excessive loading of the implant during mastication [10,11]. This leads to increased expenses for the patient as multiple appointments with the dentist are needed for implant repair or replacement [14].

Over the years, technological advances and the development of new materials, coupled with an increasing human average life expectancy, have led to more significant oral health care recurrence. Consequently, dental implant-based oral rehabilitation has become more common, with a more extensive and diverse group of patients benefiting. An effective range of solutions has been developed to address different dental problems, including using lighter implants with improved biomechanical properties and more efficient bio-integrative materials that offer a natural tooth-like aesthetic [15]. However, although dental implants can restore some degree of sensory feedback and oral function, they do not actually contribute to restoring oral proprioception due to the absence of periodontal mechanoreceptors.

### 1.2. Restoring Oral Proprioception

Neuromuscular/Transcutaneous Electrical Stimulation (NMES/TENS) biofeedback is a therapeutic technique that combines the principles of electrical stimulation and biofeedback to enhance muscle function and rehabilitation in various conditions [16,17]. Unlike other neurostimulation techniques, such as direct transcranial current stimulation, which can have mild and transient side effects, neurofeedback-based treatments are generally free from adverse effects. Regarding the mouth tract, it has been used, e.g., to improve swallowing function in patients with dysphagia [18], manage masticatory muscle activity in individuals with awake bruxism [19], and prevent temporomandibular disorders [20], resorting to NMES/TENS, or visual, auditory, and vibrational biofeedback.

The present study extends the previous research of the team presented in [13], aiming at developing an active dental prosthesis suitable to contribute to restoring the oral proprioception of people with severe tooth loss after suitable electrical stimulation. The orofacial region portrayed in this study focuses on the mandible. Consequently, the trigeminal nerve and its ramifications that innervate this region are the central nerve structures to be considered for stimulation.

Afferent nerve fibers in this nerve terminate in sensory organs (receptors), which reflect peripheral tissue stimulation [4]. In light of this, the most practical approach for evaluating sensory function in the maxillofacial region is to use the inferior alveolar nerve (IAN). That is so because stimulating the IAN peripheral receptors initiates a series of neuroelectric events that assess the functionality of the Cranial V nerve and its central connections [21]. Additionally, the procedure is performed using the most widely accepted technique, which involves observing evoked potentials (EP) [21,22,23,24,25,26,27].

The electrophysiological reaction of the nervous system to external sensory stimuli is observable as EP. The data derived from this study have the potential to enhance our understanding of the intricate neural pathways of the central and peripheral sensory nervous systems. Additionally, EP observation can offer objective and repeatable data to identify a specific pathological condition, pinpointing the precise anatomical location of the lesion and assisting in its monitoring.

Evoked potentials show low amplitudes, ranging from 0.1 to 20 μV. They are easily affected by random "noise" generated by different sources, such as electroencephalographic activity, muscle artifacts, and interferences from nearby electrical appliances. To mitigate the non-cyclical interference of the obtained signal, electrical filtering and additional average computation are usually required [23].

Although auditory, visual, and somatosensory stimuli are among the most commonly used, EPs can be activated by diverse neurostimulation modalities. The following groups have been the subject of research, each of which assesses a different sensory system: visual evoked potentials (VEPs), somatosensory evoked potentials (SEPs), and brainstem auditory-evoked potentials (BAEPs) [23]. When stimulating a branch of the trigeminal nerve, the use of SEPs, specifically trigeminal somatosensory-evoked potentials (TSEPs), is the most appropriate approach to evaluate sensory function in the maxillofacial region. It delivers precise electrical pulses to the trigeminal nerve while capturing electroencephalographic (EEG) signals, mainly in the primary somatosensory cortex, where the electrical signals from the nerve are integrated.

**Figure 1:**
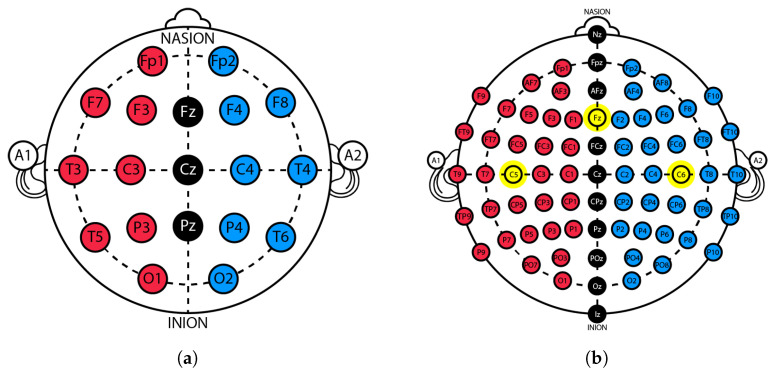
(**a**) Scalp electrodes position in the 10-20 system. (**b**) Electrodes position in the 10-10 system, highlighting C5, C6, and Fz.

Regarding EEG acquisition, the preferred system is the 10–20 system (Figure 1a), developed by the International Federation of Clinical Neurophysiology, or its variations, such as the 10-10 system. This approach, as detailed in [21], allows recording TSEPs from C5-C6 positions referenced to Fz (as shown in Figure 1b). The literature suggests that this location exhibits the most desirable levels of neuronal activity [21,22].

**Figure 2:**
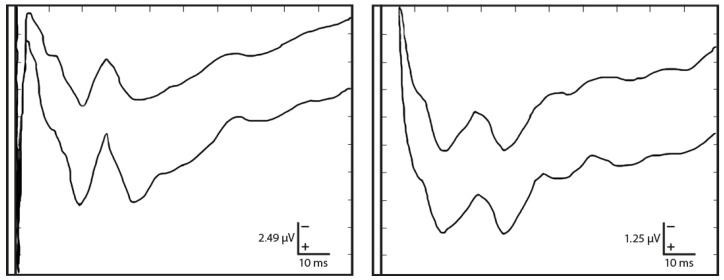
TSEP recordings from two subjects highlight the waveform shape’s stability, particularly the initial ‘W’ component. The waveforms are approximated and sketched based on [21].

As described in [21], a healthy person’s TSEPs should show four characteristic peaks, forming a sort of “W”. Their average latencies correspond to points N12, P20, N26, and P36—named after polarity (negative or positive) and latency in milliseconds after the stimulus. Their averaged peak-to-peak amplitudes typically show voltages of 1.9, 1.5, and 1.9 μV, respectively, with standard deviations of about 1 μV (Figure 2). Moreover, the response to this method is generally superior in the contralateral direction.

It should be remarked that statistically favorable results must be obtained since individuals may or may not have a response with well-defined characteristics, with some diversity in the capture sites where the signals are stronger, and the possible presence of pathologies that transfigure this response. In addition, each stimulation point can have slightly different effects on each individual since the distances between the nerves and the impedances of the tissues vary.

Different publications report the study of TSEPs concerning different pain syndromes affecting the trigeminal nerve region. These address different electrical stimulation spots, such as lips, facial skin, and gums [21,22,24,25,26,27,28,29,30,31,32]. Concerning the objectives of our study, the best non-invasive site is the gum, as it allows for better stimulation, both in terms of nerve sensitivity and the lower noise created by the electrical stimulation itself.

The present work focuses on evaluating the somatosensory feedback transmitted to the central nervous system through neural impulses generated by electrical stimulation in the gums, after the observation of the EEG response. Two methodologies were followed. The first was to ensure that the approach used for obtaining TSEPs aligns with what has been documented in the literature, reproducing the same results as previously reported.

The second concerns an innovative method, which involves modulating the stimulation frequencies with the biting force using a force sensor between the dental arches. This procedure aimed to verify the possibility of using different pulse frequencies mimicking periodontal mechanoreceptors to achieve different brain responses.

We postulated that modulating electrical stimuli frequency with bite force would induce measurable variations in TSEP latency and amplitude, distinguishing them from conventional pulse patterns, as a mechanism for encoding bite force information in neural processing. The findings of this study will contribute to evaluating the feasibility of integrating a synthetic neurostimulation system capable of delivering frequency-specific stimuli corresponding to discrete masticatory force levels, potentially enhancing sensory feedback mechanisms in oral function and prosthetic applications.

The work presented here only involves two participants due to this being a pilot study aiming at a preliminary proof of concept, to evaluate its feasibility, refine methodologies, and identify potential challenges. These regarded, namely, the design of the electronic force sensing and stimuli generating circuits, the EEG data acquisition equipment, and the environmental conditions. At this stage, it was also not feasible to involve patients, given the pilot character of the experiments. Nevertheless, ethical and safety precautions were taken into consideration. The study received formal approval, ensuring compliance with ethical standards. Participants, all of whom were members of the research team, were fully aware of this study’s objectives, procedures, potential risks, and their right to withdraw at any time before signing the consent form.

## 2. Materials and Methods

### 2.1. Traditional Trigeminal Somatosensory-Evoked Potentials

The standard procedure to generate TSEPs implies directly stimulating the trigeminal nerve or a nearby region. According to what has been reported in other works (Table 1), it was deemed appropriate to use a pulse with a frequency of 1 Hz, 200 ms duration, and an amplitude exceeding by 2–3 times the Minimum Stimulus Perception (MSP) threshold value of the subject as the stimulus. Our experimental observations have indicated that a minimum of 500 stimuli would be required to enhance the signal-to-noise ratio (SNR).

Following the studies listed in Table 1, the EEG response occurs in less than 100 ms after stimulation, so we later decided to use a frequency of 2 Hz, allowing us to reduce the duration of the acquisitions without compromising the response time. Furthermore, it was found that a stimulus amplitude of three times the MSP caused too much discomfort to the subjects, so only two times the MSP threshold was used. In addition, the pulse phase was inverted every quarter of the total number of stimulations.

The pilot study consisted of three distinct configurations for acquiring EEG signals. A fourth configuration was implemented in an innovative method, which is described in detail in the next section. Each approach has specific components and experimental conditions that stimulate particular facial regions. The systems were implemented sequentially to address limitations encountered in previous configurations.

The four setups used in this study are as follows:

- Setup 1: LTK545 3-in-1 electrostimulator from Moretti S.P.A. and eego™ mylab set from ANT Neuro (64-channel EEG cap and a 32-channel amplifier).
- Setup 2: 2100 Isolated Pulse Stimulator (A-M Systems) and eego™ mylab set from ANT Neuro (64-channel EEG cap and a 32-channel amplifier).
- Setup 3: 2100 Isolated Pulse Stimulator (A-M Systems), eego™ mini-series amplifier from ANT Neuro, and a Nihon Kohden-type EEG cap.
- Setup 4: ISO-STIM-II-Stimulus Isolator (npi electronic GmbH), eego™ mini-series amplifier from ANT Neuro, and a Nihon Kohden-type EEG cap.

Each case has unique configurations and focus areas—either lip and gingiva or gingiva stimulation alone—to investigate stimulation-triggered EEG responses with optimized reliability and precision. These are described in more detail below.

Regarding the first setup, the EEG signals were acquired using the eego™ mylab from the ANT Neuro amplifier, comprising a 64-channel EEG cap, a 32-channel amplifier, and the relevant acquisition and recording software, described further on. An LTK545 3-in-1 electrostimulator from Moretti S.P.A. was utilized for nerve stimulation. This stimulator provides a biphasic square wave with suitable waveform characteristics but does not provide a proper trigger signal synchronized with the onset of the stimulation pulse. This signal is essential to trigger the amplifier for the time onset of the event being studied, thereby allowing efficient and feasible data processing.

**Figure 3:**
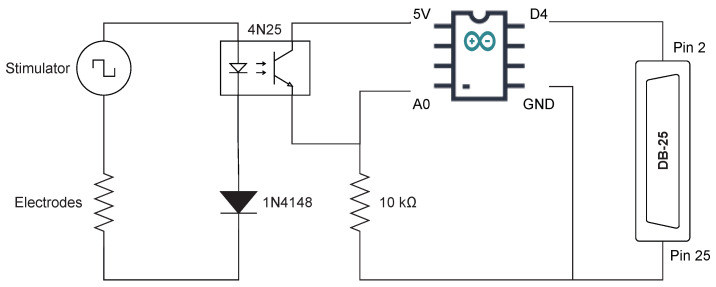
Schematic of the electronic circuit designed to trigger the amplifier for TSEP acquisition at the onset of stimulation.

To meet this objective, a circuit was devised (Figure 3) to generate a 5 V pulse with a duration of 2 ms, using a 4N25 optical coupler to achieve galvanic isolation between the stimulator and the amplifier to prevent noise interference during EEG acquisition.

**Figure 4:**
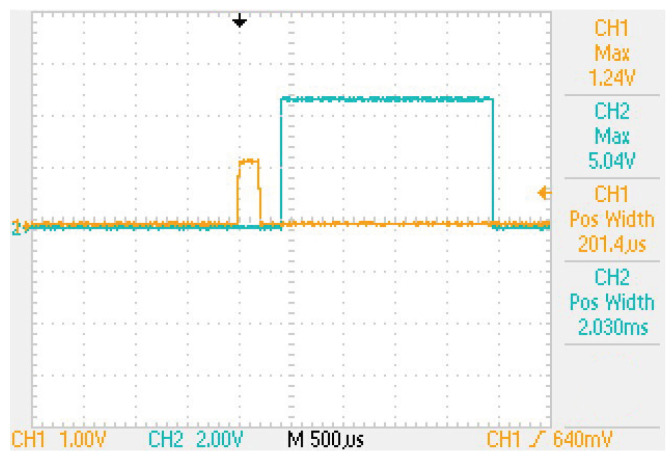
Synchronization of data acquisition with the stimulation. In orange, the signal detected at the electrodes (for 1 mA). In blue, the trigger applied to the EEG amplifier.

The left side of the schematic shows the stimulator connected to the electrodes and the stimulated tissue (represented by a resistor). During the positive half of a biphasic stimulus, the stimulation current passes through the optocoupler and a diode (1N4148). This eliminates the negative phase of the stimulus and converts it to a single-phase signal. The pulse generated at the optocoupler output is captured by an Arduino system, which generates the 2 ms trigger pulse for the data acquisition system used to capture the EEG signal (pin 2). There is a delay of about 400 μs between the onset of the stimulus and the trigger generated by the Arduino (see Figure 4). However, the resolution of the EEG data acquisition system is shorter than this time.

**Figure 5:**
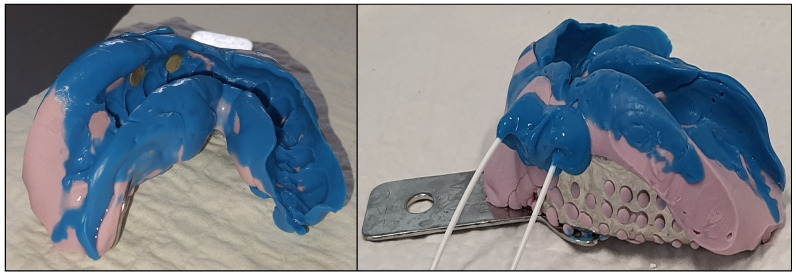
Support of the gingiva stimulation electrodes (Ag/AgCl). Shaped on the subject’s lower dental arch.

To ensure the electrodes remained fixed during gingival stimulation, a silicone cast was designed (Figure 5) to conform to the topography of the subject’s lower dental arch. This ensured that the location of the stimulation remained constant throughout all trials, eliminating additional variables. Two Ag/AgCl type electrodes were placed 1–2 cm apart, near the base of the right canine, thus close to the mental foramen nerve, as recommended in [33]. The gingival tissues exhibited a resistance of approximately 1 kΩ between the electrodes.

**Figure 6:**
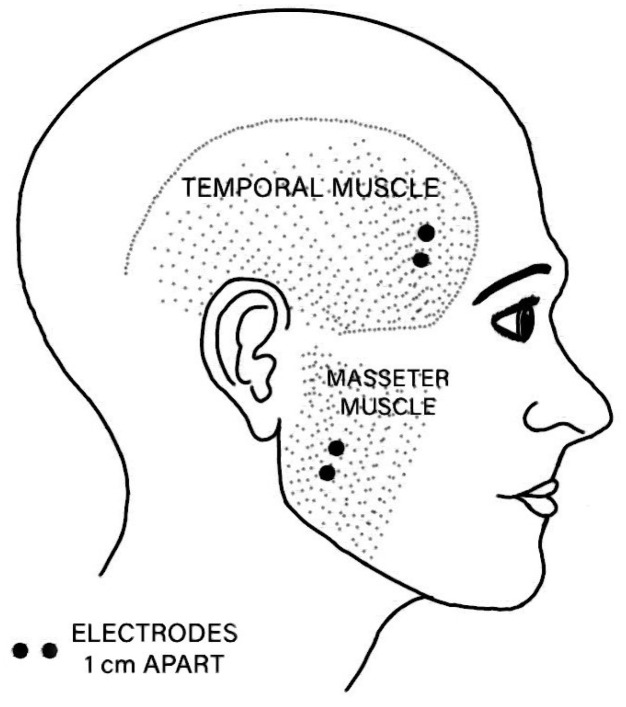
Placement of the sEMG electrodes on one side of the face during TSEP acquisitions.

To mitigate the high-amplitude artifacts observed across all EEG cap channels shortly after stimulus application—regardless of the stimulation electrodes’ placement—sEMG recordings were incorporated alongside EEG during the experiments. This approach enabled us to determine whether the artifacts were associated with cerebral signals or stimulation. The AgCl electrodes of the sEMG were placed simultaneously on the temporal and masseter muscle regions, as shown in Figure 6. Further details are provided in the subsequent sections.

The second setup was designed to overcome interferences that may be linked to the stimulator, intended for home and therapeutic use. The new equipment, a 2100 Isolated Pulse Stimulator from A-M Systems meant for laboratory applications, allowed us to obtain better results, as the amplitude and duration of the stimulation’s artifacts were significantly attenuated, allowing us to observe the desired latencies without difficulty.

The third setup was designed to improve the observed TSEP, as some artifacts could still be found superimposed on the EEG signals. Now, a single Arduino Uno functions as a pacemaker to trigger both devices at the desired frequency by sending a 5 V, 2 ms pulse through the digital pins related to the connectors. After experimentally verifying that the EEG cap being used could also affect the results of the recordings, the new acquisition system resorts to the ANT Neuro eego™ mini-series and the Nihon Kohden cap type, equipped with Ag/AgCl bridge electrodes sourced from H + H Medical Devices. This new design allows for unrestrained placement and movement of electrodes on the subject’s scalp. This freedom enables acquisitions to be performed at the locations most likely to show the signals of interest, i.e., C5 and C6, being Fz again taken as reference.

For all of the systems used, and therefore for all of the TSEP recordings, different conditions were used to experimentally identify the settings for obtaining a stimulus–response with greater precision and detail. These included establishing a connection between the participant and the floor, acquiring the EEG in an anechoic chamber, and varying the stimulation sites (on the skin under the lower lip and the gums). In addition, we examined different current intensities to detect muscle activation or lack of response. All procedures involving skin stimulation were performed with Ag/AgCl SX30 electrodes provided by Dormo.

To obtain TSEPs during the EEG acquisitions, the following protocol was adopted:

- Connect all the electronic components, including the stimulator, the circuit, the EEG cap, the amplifier, and the computer;
- Adjust the cap to the subject’s head;
- Configure the ANT Neuro recording program eego™ to measure the impedance of the EEG electrodes. Apply conductive gel to the electrodes as needed to reduce the impedance to below 20 kΩ;
- Define the software variables for each specific acquisition, namely the subject’s data, the sampling rate (2048 Hz in our case), the setup, and the amplifier;
- Select the attributes for visualizing the signal. These include the number of observable channels, the visible time window, the scale, and the band-pass filter bandwidth, which are the same as the ones used for processing, as stated below.
- Place the mold/electrodes in the orofacial area;
- Acquire the EEG signals as the stimulus is applied.

**Figure 7:**
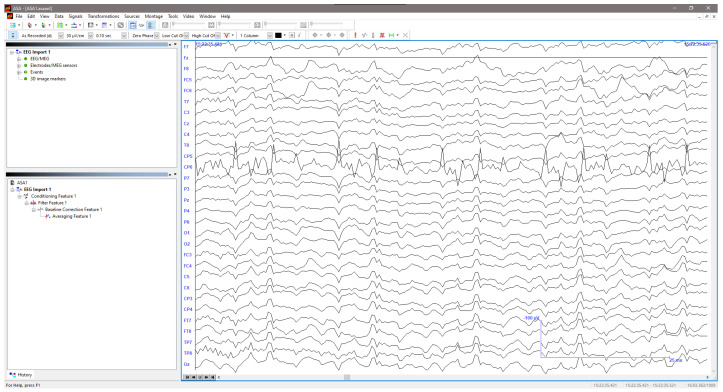
Graphical user interface of the asa™ processing program from ANT Neuro.

The acquired signals were processed with an asa™ processing program (Figure 7) using a five-step algorithm, starting with a 1–800 Hz bandwidth band-pass filtering to remove noise that could interfere with the readings. This bandwidth was selected based on the sampling rate of 2048 Hz and to avoid aliasing effects due to proximity to the Nyquist frequency.

Further manual analysis of the entire signal was carried out to exclude significant interferences, with all deviations that could negatively impact the results being eliminated. It is worth noting that the percentage of removal never exceeded 10%. Following the time location of the trigger, the entire signal was segmented into 500 ms windows. Each window spanned a duration of 150 ms before the trigger location and 350 ms after. Following segmentation, all windows were baseline-aligned to minimize fluctuations in the signal that deviated from its baseline. A 15 ms duration analysis began 20 ms before the trigger, followed by value subtraction in the existing window to obtain the final values. Finally, all windows were averaged, thereby intensifying the signal of interest while reducing random noise/interferences to obtain a 500 ms window aligned with the trigger containing the final TSEP signal.

This study phase involved a 28-year-old left-handed female with a healthy dental status. The only notable dental condition was a crown on an upper anterior tooth, which was not expected to influence the results, as all stimulations were applied to the mandibular region. No exclusion criteria were met.

### 2.2. Occluded Trigeminal Somatosensory-Evoked Potentials

Since the ultimate goal of this project is to develop a prosthetic system capable of delivering nerve-stimulating impulses at frequencies corresponding to the forces exerted during mastication, a fourth setup was devised, following the elucidations and insights previously obtained with the traditional TSEP methodology. This time, a force sensor is placed in the dental groove above the cusp of the lower left 1st molar, i.e., tooth number 19, according to the universal numbering system [34]. The sensor signal modulates the frequency of the stimulus generator based on the detected force. This add-on allowed us to assess the feasibility of reading the neural responses of the TSEPs while the subject was biting. It should also confirm whether using different frequencies within a narrow range during the same acquisition is viable, as this would account for the varying response sensitivities of the mechanical periodontal receptors.

The same equipment and EEG acquisition procedures, as defined in Section 2.1, were utilized to ensure consistency in obtaining the final results. Regarding the equipment, the ISO-STIM-II-Stimulus Isolator with bipolar output from npi electronic GmbH was used as a stimulus generator, and the eego™ mini-series amplifier with the Nihon Kohden type cap was used for EEG acquisition.

This new trial involved a different subject: a 30-year-old, right-handed male. No dental anomalies were reported or observed, and no exclusion criteria were met. The involvement of a second subject allowed us to verify the reproducibility of the methodology used in the initial test protocol with a different person and to set a baseline for the expected neural responses to traditional TSEPs. A new dental cast was created to match the subject’s lower dental arch topography, since braces must be customized for each participant.

Stimulation was applied to the left gum using a bipolar signal lasting 200 ms at a frequency of 2 Hz, with a total of 600 events recorded, set at 2.5 times the MSP threshold. Since the new stimulator delivers bipolar stimuli, manual phase inversion of the signal during the test was unnecessary.

**Figure 8:**
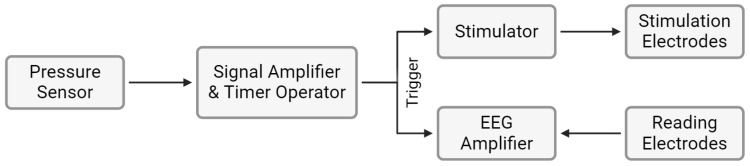
Schematic representation of the circuit used to obtain Occluded TSEPs.

Following the control experiment, the newly designed stimulation circuitry was implemented comprising the following (Figure 8):

1. Signal shaping by the sensor;
2. Signal amplification;
3. Timer operation, converting the signal into pulses at the designated frequency;
4. Signal triggering the stimulator and the amplifier.

Two tests were conducted using this configuration, with signal properties identical to the control. Stimulation was applied at approximately 0.65 Hz in the first test and 2 Hz in the second. These frequencies were chosen arbitrarily and were not intended to reflect the actual functioning of periodontal mechanoreceptors. The choice of 2 Hz as the “high” frequency was influenced by its proximity to the acquisition rate used in the previous test, as well as by limitations of the stimulation system. Higher frequencies up to approximately 10 Hz could feasibly have been employed. This is justified by the fact that the signal of interest occurs within the first 40 ms, while the baseline stabilizes effectively by 100 ms. The lower frequency corresponded to the participant’s posture, characterized by the minimal subjective force exerted between the teeth, i.e., with teeth touching in resting position, with the subject instructed to assume the centric occlusion position. The exact position was maintained for the higher frequency, though with a noticeably greater force exerted. It should be noted that the forces applied were subjective and were not recorded or converted to a standardized scale.

In summary, a frequency of 0.65 Hz produced a minimal sensor response, indicating low contact force. In contrast, the 2 Hz frequency resulted in sensor signal saturation, corresponding to maximum force application. As the objective was not to relate changes in TSEPs’ characteristics to bite force, the actual force amplitude exerted was not measured. Force variation was detected and triggered the acquisition of EEG data.

Given the extended duration of the test, which included two separate phases—first with reduced frequency and then with high frequency—it was necessary to minimize the discomfort experienced by the individual undergoing the procedure. As a result, 900 samples were collected, split into 400 for the first test and 500 for the second one. The first test was subdivided into the acquisition of 200 samples, followed by a rest period and the acquisition of the other 200 samples. On the other hand, the remaining 500 samples were taken in a single run. All data underwent the same procedure regarding data processing as described in the previous subsection.

The data were carefully processed using the same methodology outlined in the previous section.

The baseline was also a focus of analysis to enhance the understanding of the experiment. Both sets of acquisitions were processed using the same methodology, although different time windows were applied concerning the triggered events. Specifically, a window ranging from −220 ms to 100 ms, with zero aligned to the trigger point, was used. The baseline alignment correction was performed using the first 100 ms of each window. These values were selected based on the higher acquisition frequency to prevent window overlap during the averaging process.

Examining the baseline allows for assessing whether a genuine response to the provided stimuli is present. Analyzing signal variations in the absence of stimulation makes it possible to distinguish actual stimulus-related activity from spontaneous or background fluctuations.

## 3. Results

### 3.1. Traditional Trigeminal Somatosensory-Evoked Potentials

Section 2.1 describes the various approaches experimented with to determine the best response to electrical stimulation. Initially, TSEPs were obtained by stimulating the two sites usually described in the literature: the skin below the lower lip and the gums. Since the two cases were performed with similar setups, even if one was carried out in the anechoic chamber and the other in an uncontrolled environment, the respective results are presented as a single one.

The EEG readings were taken from channels around C5 and C6, specifically FC5, FC6, T7, T8, C3, C4, CP5, and CP6, as well as central channels like FpZ, Cz, Pz, and the reference Fz. A total of 200 stimulation pulses were applied without phase reversal, and the amplitudes of all pulse stimuli were set to twice the MSP threshold. Both acquisitions were made in the right head region. The trials in an anechoic chamber aimed at reducing electromagnetic interference and other external disturbances that may impact the wearer’s focus.

**Figure 9:**
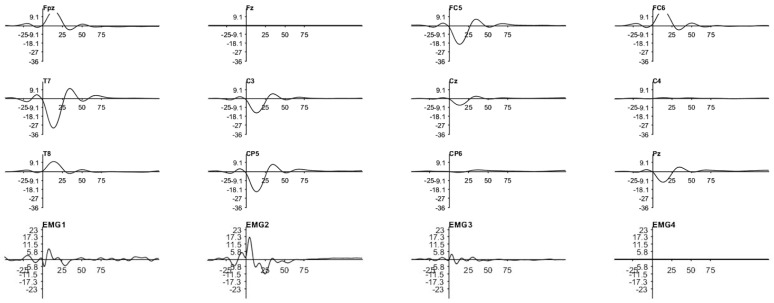
TSEP result with stimulation performed below the lower lip. Channel reading around C5 and C6 with reference in Fz. Trial performed with the first acquisition system and stimulator. Stimuli amplitude of twice the threshold current of the MSP and 200 stimulations. Y-axis in μV and X-axis in ms.

**Figure 10:**
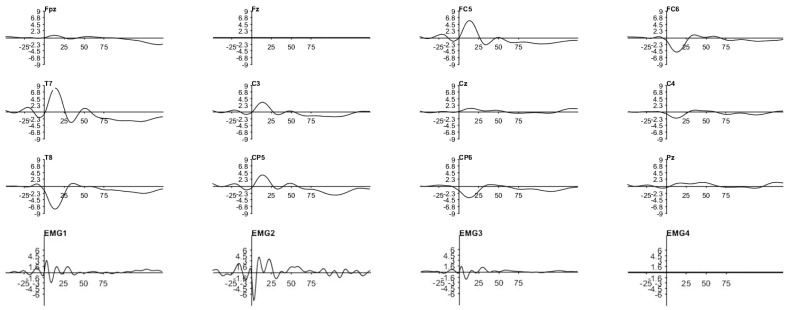
TSEP result with stimulation performed in the gingiva. Channel reading around C5 and C6 with reference in Fz. Trial performed with the first acquisition system and stimulator. Stimuli amplitude of twice the threshold current of the MSP and 200 stimulations. Y-axis in μV and X-axis in ms.

Figure 9 and Figure 10 show how a highly limited artifact corrupts the EEG across all channels in both scenarios. This corruption is due to the artifact’s large latency and amplitude compared with those reported in the literature, causing the filter response to be similarly prolonged and to suppress the entire response.

**Figure 11:**
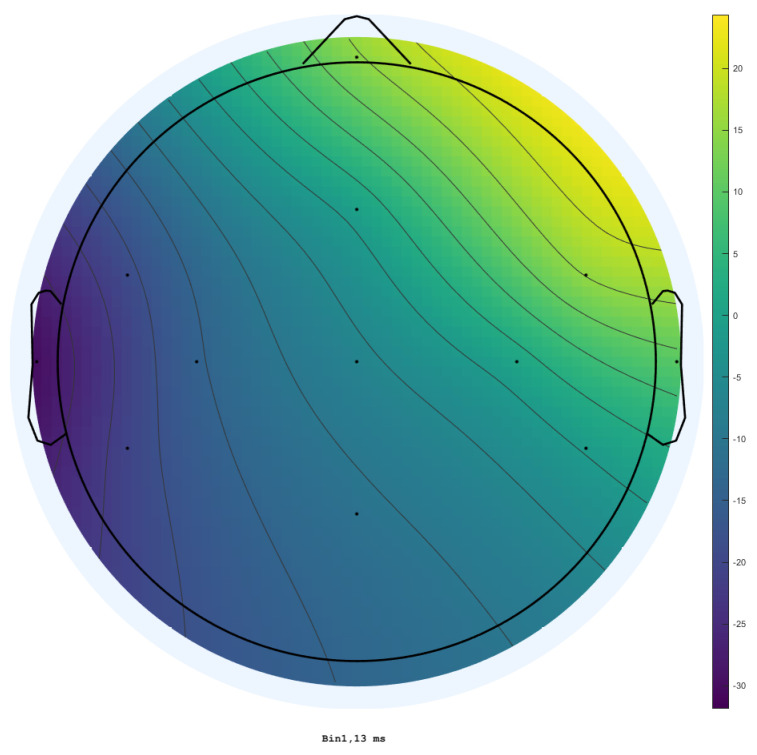
EEG scalp map gradient during TSEP acquisition, with stimulation on the right side below the lip. The relative gradient shows a diagonal voltage potential decrease from the stimulation site to the lowest intensity near the opposite ear.

Since the phase between the two cerebral hemispheres is reversed and decreases as the distance between the stimulated site and the EEG observation point increases (also seen in Figure 11), we determined that the cause of this artifact is a physiological process triggered by the stimulator. Moreover, the fact that the trials were conducted in opposite phases lends more credibility to this assumption. As can be seen by the opposing polarities in each EEG channel between these two images, only one of the polarities was used in each acquisition. Nevertheless, counteracting this process is challenging due to the numerous paths in the head through which the current can be routed.

As for the EMG data, they were acquired concurrently with the TSEPs and are shown in the bottom rows of Figure 9 and Figure 10. Table 2 describes how the name of each channel corresponds to its anatomical location. Note that the phase of the signal is not related to the phase found in the EEG, as the EMG is acquired differently.

On the other hand, it already has a direct link with the amplitude. Despite the disparities in scale between the graphs, it is evident that the EEG amplitude increases with the EMG amplitude. It is also clear that the instant immediately following the trigger shows the highest EMG amplitude. All these characteristics, in addition to the visual signal dispersed around the face, emphasize how these outcomes are connected to the stimulation procedure.

Therefore, we may conclude that the signal detected by the EEG cap is not derived, at least not exclusively, from the cerebral cortex but rather from a stimulator-induced event. Although there has been discussion about this process in some previously published literature, no workable explanation for this phenomenon has been provided.

Notwithstanding this drawback, there is an upside to this strategy, since it allowed us to confirm that the magnitude of the EMG artifact is lower in the gingival test. In addition, by averaging the data from several occurrences, one can validate that the phase inversion that occurred during the testing can be an added value, since random noise will be reduced. Likewise, we have chosen to employ more stimuli from here on as this would increase the observability of the signal of interest.

Following this phase, we changed to the second system mentioned in Section 2.1. However, since the third system is more accurate due to access to the electrodes in the preferred positions, this will be the setup used in the following tests.

The EEG artifact, also seen in the EMG signals, which was only noticeable in the first milliseconds and obscured the signal of interest, could now be further reduced due to the change to setup 3. This occurrence suggests that the phenomena induced by the previous stimulator have been significantly diminished, allowing a more precise acquisition of the TSEP signals.

Henceforth, the reading cap is configured in all trials so that C5 and C6 signals can be obtained with respect to Fz. During every trial, stimulation was performed on the inferior gum on the right side of the face, and phase reversal was implemented.

As expected, experimental verification confirmed that conducting tests in the anechoic chamber reduces electromagnetic noise, particularly in the 50 Hz range. However, it was impossible to perform additional TSEP acquisitions in the chamber due to the inability to move the stimulator being used. As a result, further tests had to be conducted in a less electromagnetically controlled environment. Therefore, it was necessary to check whether connecting the subject to the ground would reduce this noise and evaluate electromagnetic interference.

**Figure 12:**
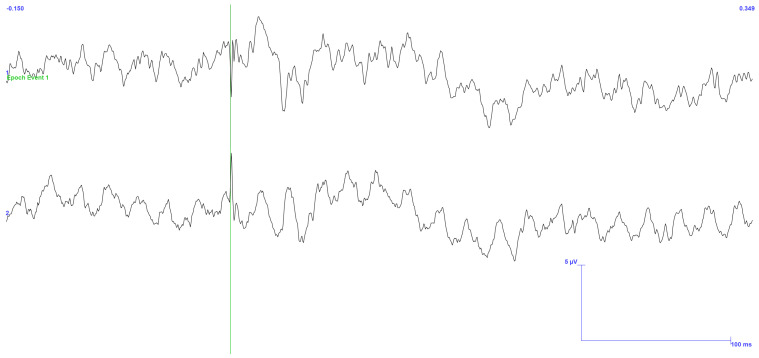
TSEP result with stimulation performed on the gingiva. Reading of channels C5 and C6 with reference in Fz. Trial performed with the third setup. Application of twice the threshold current of the MSP and 500 stimulations. Connection of the subject to the ground in a noisy environment. Green vertical line: trigger. Ch1: EEG position C6; Ch2: EEG position C5. Scale: 5 μV (y-axis), 100 ms (x-axis).

In this test, the mastoids of the subject were connected to a ground terminal, and 500 stimulations were applied at an intensity twice the MSP threshold. The periodic variation seen in the captured (Figure 12) signal, which is due to electromagnetic noise, persists even if the subject is connected to the ground. The quantity of electronic gadgets in the area is the cause of the disparity in noise consistency between the two hemispheres.

Considering this, it is also evident that this connection can be advantageous, as it allows the artifact to diminish when it discharges to the ground, leaving behind primarily the 50 Hz electromagnetic noise from the power grid. It should be noted that since the frequency of the TSEP signal is so close to 50 Hz, it is difficult to eliminate the noise digitally. Therefore, the only viable approach is to reduce the number of surrounding sources that can produce similar electromagnetic noise.

Another factor we considered essential to test was the variation of the stimulus current amplitude to confirm the reaction to the stimulation amplitude. After performing several tests, always maintaining the subject connected to ground and ensuring that non-necessary equipment was turned off, it could be concluded that a stimulation amplitude higher than the MSP is required to obtain a readable response in the EEG signal.

**Figure 13:**
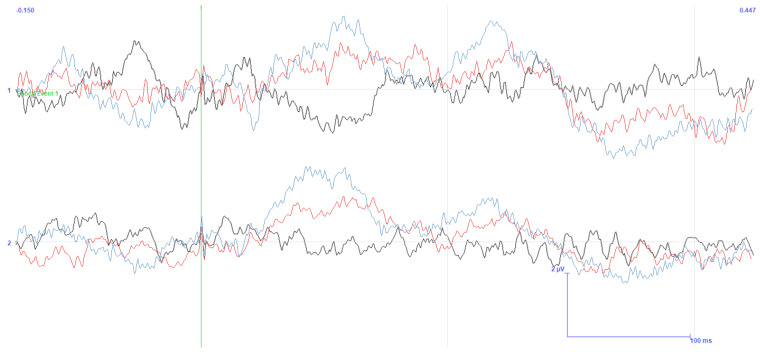
TSEP result with stimulation performed on the gingiva. Reading of channels C5 and C6 with reference in Fz. Trial performed with the third setup. TSEP conditions: 500 stimulations; black—ten times below the MSP threshold; red—equal to MSP threshold; blue—twice the MSP threshold. Green vertical line: trigger; Ch1: EEG position C6; Ch2: EEG position C5. Scale: 2 μV (y-axis), 100 ms (x-axis).

Consequently, in the following experiments, we used three current levels: below, equal to, and above the MSP level, or more specifically, 0, 1, 1, and 2 times the threshold. The results, after processing the 500 stimulations, are shown in Figure 13.

As can be observed, the response to stimulation below the threshold is essentially zero (black curve in Figure 13), and the regularity of the signal confirms that the background electromagnetic noise is predominantly present. Deviations in the signal could be attributed to trigeminal reflexes, induced muscular activity, or artifacts caused by the subject’s movement.

A decrease in electromagnetic noise is seen in the signal acquired using a stimulus with an amplitude equal to the MSP threshold value (red curve), with greater significance in the contralateral hemisphere. This phenomenon could be attributed to the stimulation of more significant physiological reactions, such as the trigeminal reflex, triggered by the electrical pulse application. However, there are still no viable data to achieve the desired outcome in the area of interest.

Lastly, a response (blue curve in Figure 13) is shown in the first 50 milliseconds following the trigger for the stimulus with the highest amplitude. Note that the acquisition of this signal shares the same physiological reactions as the previous one, although with a higher response.

These findings suggest that a value greater than the MSP threshold must be applied when identifying the response waveform to the stimulus under analysis.

**Figure 14:**
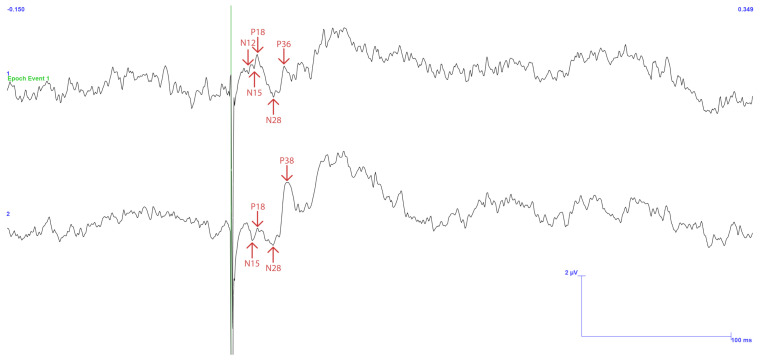
TSEP result with stimulation performed on the gingiva. Reading of channels C5 and C6 with reference in Fz. Trial performed with the third setup. Application of twice the threshold current of the MSP and 600 stimulations. Green vertical line: trigger. Ch1: EEG position C6; Ch2: EEG position C5. Scale: 2 μV (y-axis), 100 ms (x-axis).

Once we performed these tests to determine the best conditions to acquire TSEPs without raising the subject’s discomfort, we proceeded to the final acquisition. The results can be seen in Figure 14. It should be noted that the subject should be comfortable during these trials since it lessens the likelihood that noise and artifacts will affect the EEG signal.

A total of 548 samples of 600 stimulations were analyzed and delivered during this acquisition. The graph is presented unconventionally, with the positive axis pointing upwards. However, the labels at the points of interest remain consistent, allowing for direct comparison. The EEG result indicates that the delivery of the electrical stimulation triggered a physiological mechanism that produced an initial peak near the trigger. However, its brief latency can be ignored because it allows for a direct reading of the relevant signal.

As far as the literature is concerned, as referred to in Section 1, four characteristic peaks can be considered in TSEPs that form a ‘w’ shape. The latencies of their mean values are associated with points N12, P20, N26, and P36. Peak-to-peak amplitudes correspond on average to 1.9, 1.5, and 1.9μV, respectively, with standard deviations close to 1 μV.

Concerning the results obtained in channels 1 and 2, i.e., C6 and C5, respectively, the signals show latencies similar to those previously identified (see Table 3). However, their amplitudes vary more significantly.

In both channels, the latencies of points P20 and N26 deviate by 2 ms in opposite directions, with peaks occurring at points P18 and N28. Nonetheless, this deviation is within the standard deviation reported in the literature. Point P36 shows the same time coordinate in channel 1, but in channel 2, it is deviated by 2 ms (P38), which is not significant due to the reason previously mentioned. Finally, point N12 already possesses other features. In channel 2, there is a deviation of 15 ms, which differs from the formats identified in the literature. Moreover, in channel 1, there are two distinct protrusions, N12 and N13, which are close to each other.

These discrepancies may not be representative given the low number of samples from different subjects. Except for the first, the peak-to-peak amplitudes fall within the range of values stated above. This occurrence may be connected to the quantity of stimuli and the number of subjects. Regardless, the contralateral side stands out for having a larger peak-to-peak amplitude than the ipsilateral side, which aligns with descriptions in the literature of this domain.

Another aspect to notice is that immediately following the region of interest, there is a subsequent divergence from the baseline that is present throughout all acquisitions and ranges from about 50 ms to 300 ms. The trigeminal reflexes may be connected to this noticeable divergence, which may even start during the cerebral cortex’s stimulus–response process. According to published research, direct motor responses can mask brain activity during the first 10 ms of acquisition, whereas various trigeminal reflexes can attenuate the signal afterward [24].

Conclusively, the results gathered here show that the materials and techniques adopted in this work allow for the observation of TSEPs and the carrying out of the corresponding processing.

### 3.2. Occluded Trigeminal Somatosensory-Evoked Potentials

As mentioned in Section 2.2, since changing the subject from the initial tests was necessary, the control methodology was used again to compare procedures. Thus, results were first captured without using the modulation of the stimuli frequency by the force sensing.

**Figure 15:**
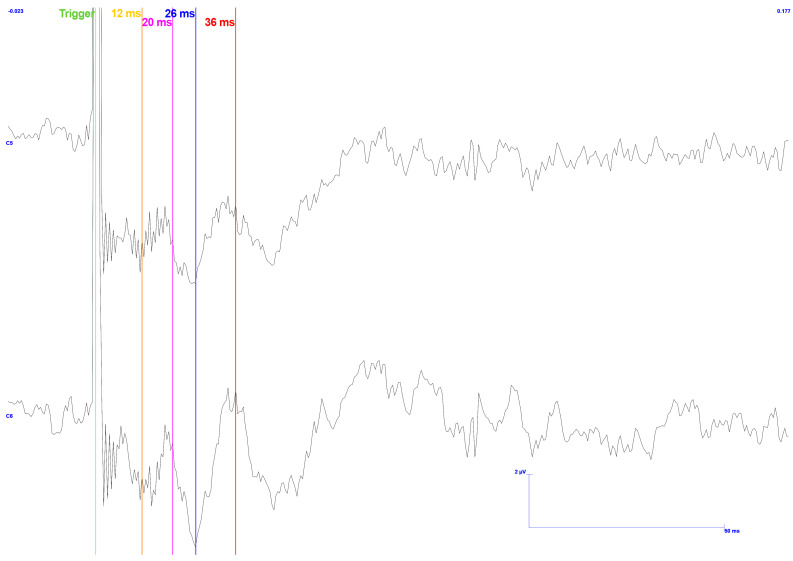
TSEP control results with stimulation performed on the lower and left side of the gingiva—reading of channels C5 top and C6 bottom with reference in Fz. Vertical bars with corresponding markers represent the expected latencies and trigger identification. Scale: 2 μV (y-axis), 50 ms (x-axis).

Figure 15 displays the signals acquired in C5 and C6 electrodes in the upper and lower regions, respectively. The average of 598 pulses is presented after removing only two events with exacerbated noise and processing the remnant. Furthermore, the anticipated latencies are displayed in a vertical bar format based on previous research and findings, as mentioned above.

As in the previous result, this one displays a pulse response directly linked to the stimulation input signal immediately following the trigger signal. The pulse response then decays, as represented by the ripple. However, these details can be disregarded as they fall outside the region of interest.

Regarding latencies, they broadly match the expected values, specifically points N12, P20, N26, and P36, with only a deviation of up to 2 ms in the peaks. In this study, this deviation is of negligible importance.

The C6 channel outperforms the C5 channel in terms of amplitude. Given that stimulation was applied to the left side of the gingiva, the outcome is consistent with what was initially foreseen. One can observe that the signals fall within this range, although they are pretty close to the upper limits, by using the peak-to-peak values 1.9, 1.5, and 1.9 μV, with a variance of up to 1 μV, as noted in the literature. So much so that the final two peaks in the C6 signal, N26 and P36, exhibit remarkably large amplitudes. As the previous results show, the first value exceeds the expected limit by 1.6 times. The second value is twice as high.

The acquisition efficiency may be attributed to the notable quality of approximately 99.7% of the signal, mainly unaffected by noise. This absence of interference encompasses both electromagnetic disturbances and dynamic noise arising from subject movement.

Despite variations in certain aspects of the signal’s amplitude, this type of study underscores the significance of latency. It is a crucial metric for confirming the anticipated ’w’ shape. In summary, the investigation of TSEPs has yielded the expected response, validating its efficacy.

With the control objective attained, the subsequent segment presents and elucidates the results obtained from the experimental phase of this methodology. Initially, the focus lies on the low-pressure acquisition and then on the situation where the jaws are clenched.

It is known a priori that the periodontal mechanoreceptors of the individual will activate due to their overall good dental health, eventually influencing the results as soon as pressure is applied to the dental arches. In any case, the signals will be evaluated using established metrics, considering the possibility of this outcome.

**Figure 16:**
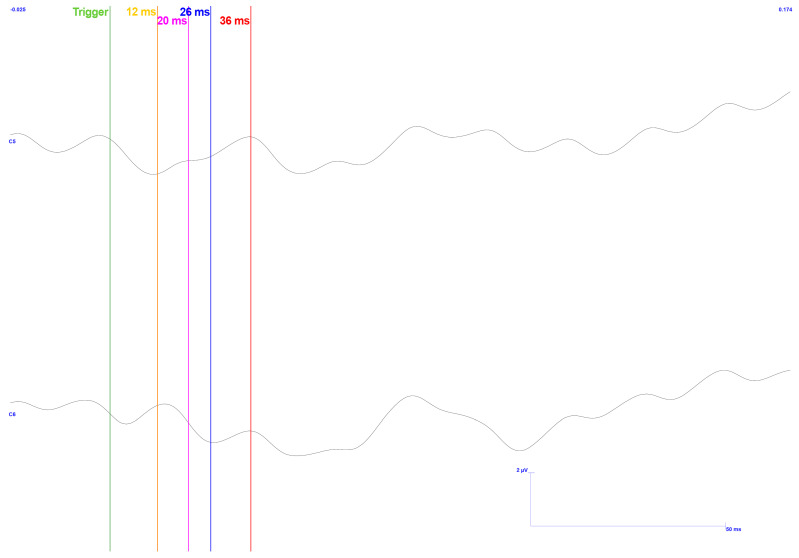
TSEP results with stimulation performed on the lower and left side of the gingiva—reading of channels C5 and C6 with reference in Fz. Low pressure exerted. Vertical bars with corresponding markers represent the expected latencies and trigger identification. Scale: 2 μV (y-axis), 50 ms (x-axis).

The acquisition of the first trial mentioned is represented in Figure 16.

As in the previous results, this representation shows the latencies of interest on both electrodes. All the points correspond to the expected concavity inversions in the ipsilateral signal. However, only the last two have this characteristic in the contralateral signal. Although the first latency is close to the reference, it is delayed by 3 ms and does not have the demanded concavity, i.e., it does not correspond to N12. The third control latency does not correspond to the acquired signal.

In terms of amplitude, only the first two peaks show values within the mentioned limits, provided they are considered adjacent latencies. Otherwise, in both signals, the voltage difference between the peaks is insufficient to meet these limits.

It should be noted that only about 260 of the 400 samples reached the final processing stages, as the rest were corrupted by noise. This amount is relatively low for a complete study of this phenomenon compared with previous ones. It is important to note that, following data processing, all signals exhibit a background frequency component approximately equal to the operating frequency of the system’s power supply (50 Hz).

**Figure 17:**
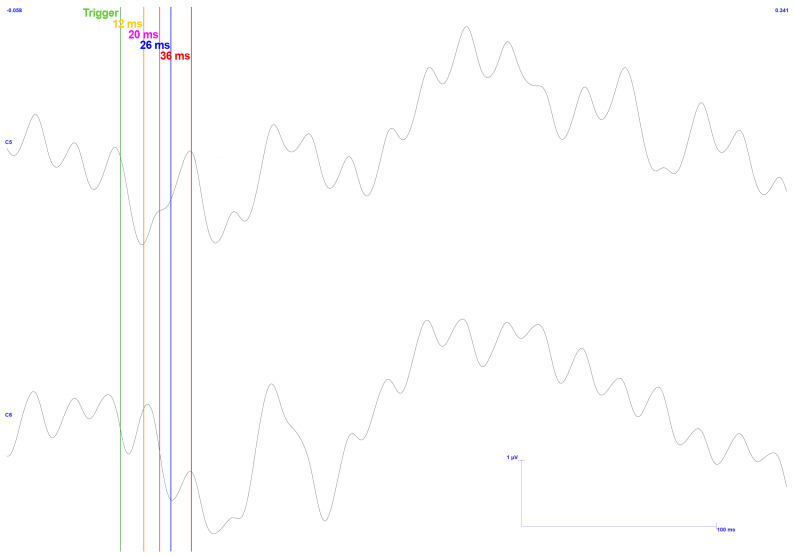
TSEP results with stimulation performed on the lower and left side of the gingiva—reading of channels C5 and C6 with reference in Fz. Low pressure exerted. Vertical bars with corresponding markers represent the expected latencies and trigger identification. Increased scale. Scale: 1 μV (y-axis), 100 ms (x-axis).

Due to the experimental nature of this methodology, we decided to change the scale of this information search window. This development led to the results shown in Figure 17. Here, we see the same information as in the previous image but with a different scaling. The vertical axis (voltage) has halved, and the horizontal axis (time) has doubled.

By resizing the signal visualization window, we can see a type of response in the neural signal, particularly on C6, i.e., the contralateral side. A structure with characteristics identical to the control test can even be seen in a particular ’w’ shape. However, it has latencies and amplitudes outside the anticipated range. This shape can be seen in peaks N26, P36, N48, and P78. It should be noted that after this response, there is a deviation from the signal baseline, as in the control test.

As this result is found with periodic noise, specific details may not correspond precisely to the neural response to the stimulation we want to find. As a result, the values, with the main emphasis on latencies, may deviate.

**Figure 18:**
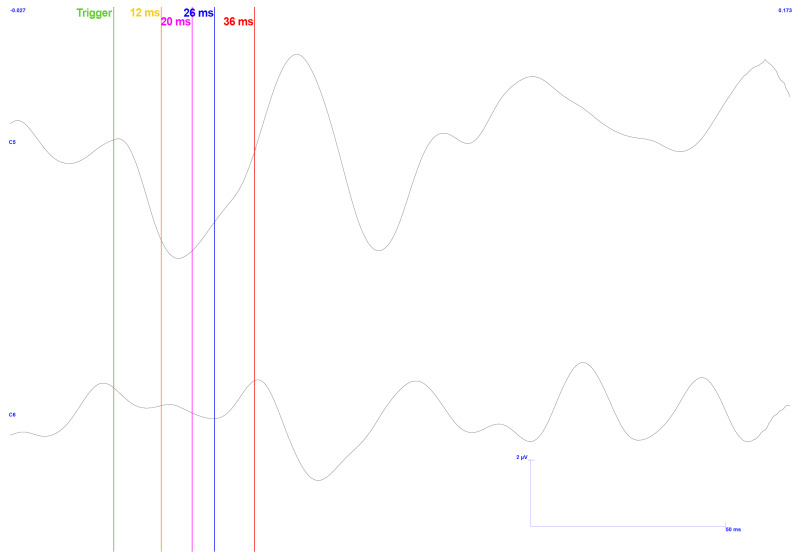
TSEP results with stimulation performed on the lower and left side of the gingiva—reading of channels C5 and C6 with reference in Fz. High pressure exerted. Vertical bars with corresponding markers represent the expected latencies and trigger identification. Scale: 2 μV (y-axis), 50 ms (x-axis).

Following this, Figure 18 illustrates the signals captured when the subject exerts a significant force on the sensor.

Regarding latencies, those in channel C6 closely resemble those of the previous result, exhibiting peak deviations of equal magnitude at this resolution. Furthermore, concavity inversion is present in the first peak, along with the absence of the second peak. On the ipsilateral side, latencies of interest can hardly be distinguished, apart from the presence of an N17.

Considering the signals’ amplitudes, the high peak-to-peak voltage of C5 compared with C6 is visible. However, this phenomenon should not occur in these TSEPs in the first place, with a control test proving otherwise. In any case, the ipsilateral values are too high compared with those seen in the literature and the control. C6, on the other hand, shows values that are too low, except P36, which is in the lower limit.

After processing this data, the final result consists of approximately 380 samples from the original 500. Although it was expected that the higher force test would produce more noise than the lower force test, resulting in the initial exclusion of signal segments, this was not observed, resulting in a lower percentage of exclusion.

**Figure 19:**
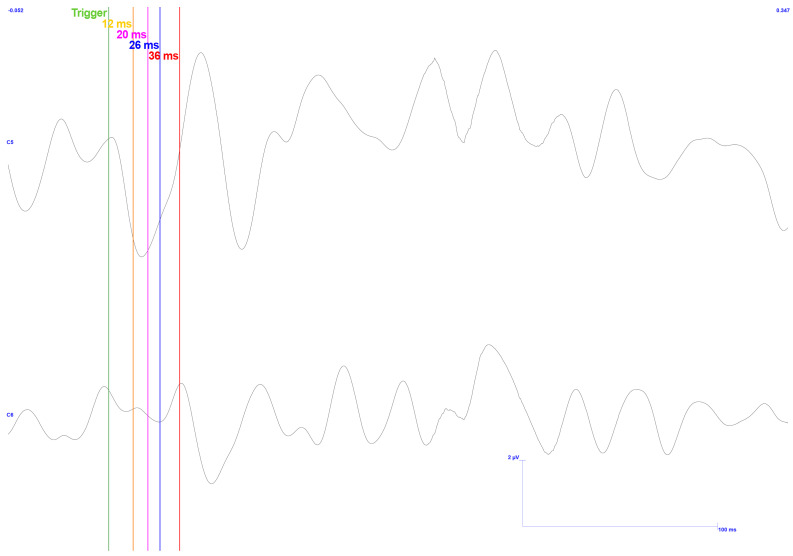
TSEP results with stimulation performed on the lower and left side of the gingiva—reading of channels C5 and C6 with reference in Fz. High pressure was exerted. Vertical bars with corresponding markers represent the expected latencies and trigger identification. Increased scale. Scale: 2 μV (y-axis), 100 ms (x-axis).

Like the previous result, background noise can be seen throughout the signal, as shown in Figure 19.

This resizing of the visualization window only involves doubling the time scale. The amplitude remains unchanged because the C5 signal extends beyond the graph.

Although some deviation can be identified and may be related to a response, these results are not explicit. The concavities are inverted, the latencies of these deviations are different from expected, and the signal from C5 is considerably higher than C6 at this location.

Moreover, this is the only result that shows minimal deviation from the baseline following a potential neuronal response, remaining closely aligned with it despite the ongoing interference from the intruding frequency.

As previously mentioned in the previous section, the same signals were reprocessed with greater emphasis on the baseline by adjusting the analysis window. This shift allows for improved observation of potential stimulus-related responses, offering a clearer understanding of their presence in the data.

For a response to be considered meaningful, it must emerge clearly from the baseline, which is expected to appear relatively flat in the absence of any stimulus-related activity. This distinction should be visible in the temporal alignment of the data, with the baseline occurring before the trigger point and any potential response appearing after the respective stimulus latency delay.

**Figure 20:**
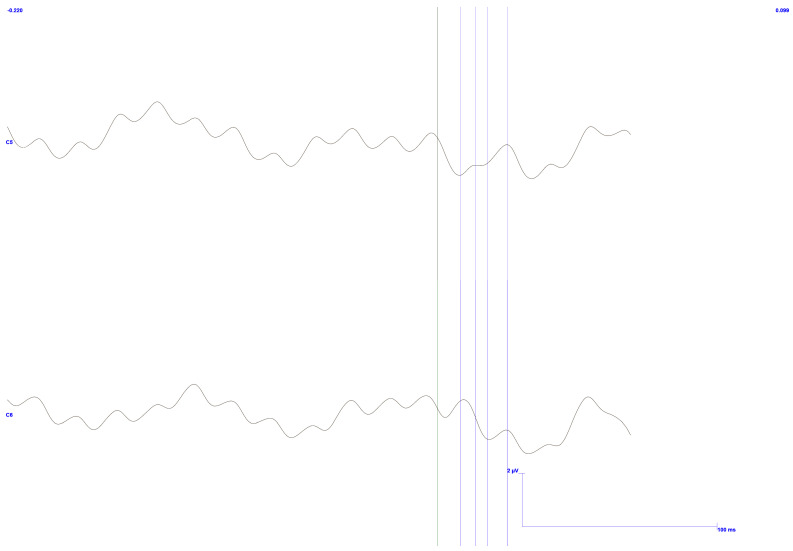
TSEP results with stimulation performed on the lower and left side of the gingiva—reading of channels C5 and C6 with reference in Fz. Low pressure is exerted on teeth. Baseline focused. The vertical bars with the corresponding markers represent the expected latencies and trigger identification. Scale: 2 μV (y-axis), 100 ms (x-axis).

**Figure 21:**
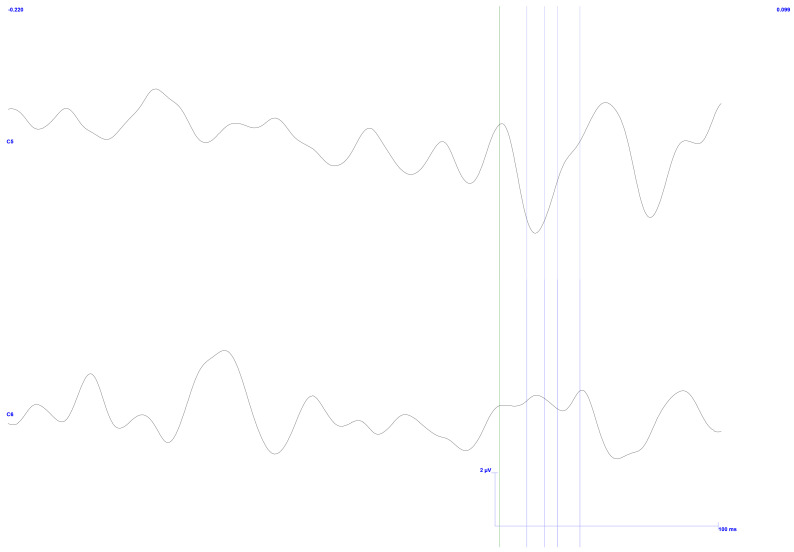
TSEP results with stimulation performed on the lower and left side of the gingiva—reading of channels C5 and C6 with reference in Fz. High pressure is exerted on teeth. Baseline focused. The vertical bars with the corresponding markers represent the expected latencies and trigger identification. Scale: 2 μV (y-axis), 100 ms (x-axis).

The results related to these modifications can be seen in Figure 20 and Figure 21, corresponding to low pressure and high pressure, respectively.

As expected, both results exhibit background noise at 50 Hz. In line with expectations, the higher pressure response shows more significant fluctuation throughout the signal. This is likely due to noise introduced during acquisition, resulting from the force exerted by the muscles. Furthermore, it may also reflect the subject’s fatigue, which can lead to variations in force and an increase in relative postural movement. Moreover, the force applied to the mouthpiece may contribute to increased discomfort for the user.

Besides noise, Figure 20 clearly illustrates a noticeable distinction between the baseline and the signal after applying the stimulus. The baseline remains relatively flat before the stimulus, indicating minimal variation. However, once the stimulus is applied, an apparent variation in the signal is introduced, marking a departure from the baseline. This change in the signal indicates the response to the stimulus, providing valuable insight into how the system reacts to the applied force.

Regarding Figure 21, a variation can also be observed in both channels compared with the period before the trigger, with a more pronounced effect in the C5 channel. Such behavior is consistent with the previously mentioned results, as the acquired signal is the same.

Focusing on the C6 channel, a significant artifact is observed in the baseline region, distorting the expected signal further. This anomaly may be related to previously mentioned factors, such as noise interference or signal distortion, which could be influenced by external variables or technical limitations in the acquisition process. However, despite these considerations, it cannot be definitively linked to any single cause, and further investigation would be necessary to clarify its origin and potential impact on the results.

## 4. Discussion

This pilot study aims to identify the optimal characteristics of gingival electrical stimulation capable of mimicking the function of periodontal mechanoreceptors. Verifying this functionality and determining the proper implementation conditions is the first step in developing active prostheses that help restore dental proprioception in people with missing teeth, particularly edentulous patients. The obtained results do not allow us to draw final conclusions but provide preliminary good indications on the stimuli duration and amplitude.

One can see that the trials performed with dental occlusion suggest a different context for neural pathway stimulation, resulting in altered brain activity related to sensory processing and motor control.

Once the results obtained following the traditional methodology are comparable to those reported in other studies, we can be confident in the results obtained within the preliminary control trial. Thus, it can be stated that the methods and equipment used in the trials with occlusion are viable for acquiring TSEPs with a high degree of confidence. Although the results could be more statistically robust were a higher number of subjects involved, the common underlying trial conditions also provide confidence in these previously unreported results.

The different latency values present in the evoked potentials after the stimulation associated with a low bite clenching force can be related to a specific response, namely the lengthening of the reaction to the stimulus. The offset to the trigger and the temporal spacing of this response are greater than those observed in the control response. However, as it is visibly stronger on the contralateral side, it may be directly related to the natural response. We understand that since the contact between teeth is always present during the tests, the periodontal mechanoreceptors’ response is constantly activated. A slight change in the pressure exerted during acquisitions occurs, even if minimal. Thus, there is a constant neuronal response since mechanoreceptors can detect these variations. This phenomenon is further emphasized by the results processed with a focus on the baseline, which shows the sustained nature of this neuronal activity.

Hence, the natural and the electrical-stimulation-related reactions might overlap, wherein the stimulation is directly linked to the trigger, being the response associated with the natural phenomenon diffused throughout the test. Nevertheless, once permanent teeth contact is established, it can be inferred that this response is consistently applied and amplified with stimulation, resulting in an increased amplitude upon processing this signal. Consequently, a response to the stimulus, distinct from the baseline (i.e., prior to stimulation), is observed, albeit at higher latencies.

Concerning the higher bite clenching force test, its characteristics pose challenges for assessment, regardless of the scale utilized. Initially, it was anticipated that heightened muscle activation would impact TSEP readings as it engages muscles adjacent to the reading electrodes. However, several factors within the signals complicate the assessment of this phenomenon, including the amplitude superiority of the ipsilateral over the contralateral, phase opposition between signals, namely near the region of interest, and the introduction of background frequency into the signal.

The amplitude discrepancy could be due to multiple electrical wires exiting the mouthpiece and extending outward from the left side of the mouth. This arrangement could become uncomfortable throughout the test, causing the subjects to adapt by exerting more force on the left facial muscles. In addition, increased pressure on the mouthpiece could change the contact impedance between the electrodes and the gums. This change in impedance could affect the perception and amplitude of the delivered stimulus, potentially resulting in involuntary activation of the left maxillofacial muscles.

Since a deviation from baseline signals was observed, this test provides evidence of a response, but it cannot be concluded that it is solely neural. The observed deviation suggests a potential response that could be more complex, involving different patterns of activity from the brain’s central integration mechanisms. In addition, when higher pressures are applied, an increase in variables may introduce responses that are not exclusively neural, potentially confounding the results.

In conclusion, it is essential to perform new tests with a larger sample size in order to increase the certainty of these conclusions. The low force test suggests a positive neuronal response to the stimuli applied, although the latencies observed are different from those reported in the literature. It is also essential to carry out high-pressure tests to determine whether the characteristics identified persist under these conditions. In addition, it would be an important step to assess whether the stimulus applied in both tests remains consistently at 2.5 times the MSP, even with the current modifications.

As the new methodology mirrors the control setup except for the sensor component system, it can be inferred that the additional frequency component corresponding to the 50 Hz noise is due to interference from the sensor system.

Future tests should take this into account and aim to mitigate any interference it may cause. These new tests should be carried out on edentulous patients wearing custom-made active prostheses, on the one hand, to confirm the absence of natural neural activity due to natural mechanoreceptors and, on the other hand, to avoid the discomfort of an apparatus connected to external equipment. These conditions should contribute to removing artifacts. Additionally, with a higher number of volunteers, control and test groups can be established.

Taking all factors into consideration, it can be concluded that there is a response to gingival stimulation and that the development and evaluation of the proposed innovative prostheses are feasible.

## Figures and Tables

**Table 1:** Information compiled from other published SEP studies. Data on stimulation are obtained for a possible comparison of criteria. Blank information (‘-’) means the cited paper lacks information about these parameters. MSP—Minimum Stimulus Perception/Sensory Threshold.

Wave Type	Signal	Intensity (mA)	Pulse Width (ms)	Frequency (Hz)	Quantity to Average	Noise Reduction	Study
Square	Monophasic	4–6.5	0.2	0.7	200	-	[21]
-	-	3–5 times MSP	-	-	50	-	[27]
-	-	3–4 times MSP	<1	0.9–3.5	64–1024	Phase reversal	[22]
Square	Monophasic	Slightly above MSP (const. V)	0.2–0.5	1–2.5	526	Phase reversal (each 64 stimulations)	[26]
-	-	<10	0.1	2	-	-	[25]
-	Monophasic	2–3 times MSP	-	3–5	500	GND between stimulation and recording sites; Repeat the process twice	[24]
Square	Biphasic	3 times MSP (const. A)	0.5	1	600	Biphasic	[31]
-	-	-	0.2–0.3	-	-	-	[32]

**Table 2:** Correspondence of the EMG channels with the anatomical site.

EMG Channel	Anatomical Site
EMG1	Right temporal muscle
EMG2	Right masseter muscle
EMG3	Left temporal muscle
EMG4	Left masseter muscle

**Table 3:** Comparison of latencies between the results obtained in study [21] and in this study.

Literature Latencies (ms) [21]	Result Latencies (ms)	
	EEG Position C6	EEG Position C5
12	12/15	15
20	18	18
26	28	28
36	36	38

## Data Availability

The datasets presented in this article are not readily available because all information collected during the trials has been kept strictly confidential. Requests to access the datasets should be directed to the first author.

## References

[1] Baiju R., Peter E., Varghese N., Sivaram R. (2017). Oral health and quality of life: Current concepts. J. Clin. Diagn. Res..

[2] Trulsson M., Van der Bilt A., Carlsson G., Gotfredsen K., Larsson P., Müller F., Sessle B., Svensson P. (2012). From brain to bridge: Masticatory function and dental implants. J. Oral Rehabil..

[3] Johnsen S.E. (2005). Human Periodontal Mechanoreceptors: Functional Properties and Role in Jaw Motor Control.

[4] Sessle B. (2006). Mechanisms of oral somatosensory and motor functions and their clinical correlates. J. Oral Rehabil..

[5] Marchena-Rodríguez A., Moreno-Morales N., Ramírez-Parga E., Labajo-Manzanares M.T., Luque-Suárez A., Gijon-Nogueron G. (2018). Relationship between foot posture and dental malocclusions in children aged 6 to 9 years: A cross-sectional study. Medicine.

[6] Shi J., Leng W., Zhao L., Deng C., Xu C., Wang J., Wang Y., Peng X. (2018). Tooth loss and cancer risk: A dose - response meta analysis of prospective cohort studies. Oncotarget.

[7] Fang W.L., Jiang M.J., Gu B.B., Wei Y.M., Fan S.N., Liao W., Zheng Y.Q., Liao S.W., Xiong Y., Li Y. (2018). Tooth loss as a risk factor for dementia: Systematic review and meta-analysis of 21 observational studies. BMC Psychiatry.

[8] Trulsson M. (2006). Sensory-motor function of human periodontal mechanoreceptors. J. Oral Rehabil..

[9] Hunter E., Congdon N., de Moura Brito L., McKenna G., Petrauskiene E., Rodrigues Leles C., Tsakos G., Woodside J., Virgili G., Piyasena P. (2023). The Global Impact of Edentulism: A Systematic Review. Eur. J. Public Health.

[10] Misch C.E. (2014). Dental Implant Prosthetics-E-Book.

[11] Goodacre C.J., Bernal G., Rungcharassaeng K., Kan J.Y. (2003). Clinical complications with implants and implant prostheses. J. Prosthet. Dent..

[12] Helkimo M. (1974). Studies on function and dysfunction of the masticatory system: IV. Age and sex distribution of symptoms of dysfunction of the masticatory system in Lapps in the north of Finland. Acta Odontol. Scand..

[13] da Silva J.M., Cerrone I., Malágon D., Marinho J., Mundy S., Gaspar J., Mendes J.G. (2020). A Smart Dental Prosthesis to Restore Dental Proprioceptivity. Proceedings of the 2020 XXXV Conference on Design of Circuits and Integrated Systems (DCIS).

[14] Levin L. (2008). Dealing with dental implant failures. J. Appl. Oral Sci..

[15] Porter J.A., Von Fraunhofer J.A. (2005). Success or failure of dental implants? A literature review with treatment considerations. Gen. Dent..

[16] Giggins O., Persson U., Caulfield B. (2013). Biofeedback in rehabilitation. J. Neuroeng. Rehabil..

[17] Toledo-Peral C.L., Vega-Martínez G., Mercado-Gutiérrez J.A., Rodríguez-Reyes G., Vera-Hernández A., Leija-Salas L., Gutiérrez-Martínez J. (2022). Virtual/Augmented Reality for Rehabilitation Applications Using Electromyography as Control/Biofeedback: Systematic Literature Review. Electronics.

[18] Huckabee M.L., Mills M., Flynn R., Doeltgen S. (2023). The Evolution of Swallowing Rehabilitation and Emergence of Biofeedback Modalities. Curr. Otorhinolaryngol. Rep..

[19] Vieira M.d.A., Oliveira-Souza A.I.S.d., Hahn G., Bähr L., Armijo-Olivo S., Ferreira A.P.d.L. (2023). Effectiveness of Biofeedback in Individuals with Awake Bruxism Compared to Other Types of Treatment: A Systematic Review. Int. J. Environ. Res. Public Health.

[20] Florjanski W., Malysa A., Orzeszek S., Smardz J., Olchowy A., Paradowska-Stolarz A., Wieckiewicz M. (2019). Evaluation of Biofeedback Usefulness in Masticatory Muscle Activity Management—A Systematic Review. J. Clin. Med..

[21] Arcuri C., Muzzi F., Docimo R., Fusco E., Pauri F., Rossini P.M. (2006). Somatosensory evoked potentials of inferior alveolar nerve. J. Oral Maxillofac. Surg..

[22] Bennett A., Wastell D., Barker G., Blackburn C., Rood J. (1987). Trigeminal somatosensory evoked potentials: A review of the literature as applicable to oral dysaesthesias. Int. J. Oral Maxillofac. Surg..

[23] Waldman H.J. (2007). Evoked potential testing. Pain Management.

[24] Cruccu G., Aminoff M., Curio G., Guerit J., Kakigi R., Mauguiere F., Rossini P., Treede R.D., Garcia-Larrea L. (2008). Recommendations for the clinical use of somatosensory-evoked potentials. Clin. Neurophysiol..

[25] Barker G., Bennett A., Wastell D. (1987). Applications of trigeminal somatosensory evoked potentials (TSEPs) in oral and maxillofacial surgery. Br. J. Oral Maxillofac. Surg..

[26] Badr G.G., Hanner P., Edström S. (1983). Cortical evoked potentials in response to trigeminus nerve stimulation in humans. Clin. Electroencephalogr..

[27] Wang K., Xu W. (2011). Trigeminal somatosensory evoked potential in inferior alveolar nerve damage assessment. J. Brain Sci..

[28] Cruccu G., Truini A. (2006). Diseases of cranial nerves and brainstem. Handbook of Clinical Neurophysiology.

[29] Villanueva J., Viñuales M., Montes C. (1998). Somatosensory trigeminal evoked potentials after gingival stimulation of the mental nerve. Normal values. Rev. De Neurol..

[30] Stöhr M., Petruch F., Scheglmann K. (1981). Somatosensory evoked potentials following trigeminal nerve stimulation in trigeminal neuralgia. Ann. Neurol. Off. J. Am. Neurol. Assoc. Child Neurol. Soc..

[31] Maezawa H., Hirai Y., Shiraishi H., Funahashi M. (2014). Somatosensory evoked magnetic fields following tongue and hard palate stimulation on the preferred chewing side. J. Neurol. Sci..

[32] Singer B. (1987). Functional electrical stimulation of the extremities in the neurological patient: A review. Aust. J. Physiother..

[33] Polk B.J., Stelzenmuller A., Mijares G., MacCrehan W., Gaitan M. (2006). Ag/AgCl microelectrodes with improved stability for microfluidics. Sens. Actuators B Chem..

[34] American Dental Association (1999). Current Dental Terminology.

